# Impact of Astrocyte Depletion upon Inflammation and Demyelination in a Murine Animal Model of Multiple Sclerosis

**DOI:** 10.3390/ijms20163922

**Published:** 2019-08-12

**Authors:** Lisa Allnoch, Wolfgang Baumgärtner, Florian Hansmann

**Affiliations:** 1 Department of Pathology, University of Veterinary Medicine Hannover, 30559 Hannover, Germany; 2 Center for Systems Neuroscience, 30559 Hannover, Germany

**Keywords:** aquaporin 4, astrocytes, blood-spinal cord barrier, glial fibrillary acidic protein, *GFAP-thymidine kinase* transgenic *SJL* mice, Theiler’s murine encephalomyelitis

## Abstract

Astrocytes play a key role in demyelinating diseases, like multiple sclerosis (MS), although many of their functions remain unknown. The aim of this study was to investigate the impact of astrocyte depletion upon de- and remyelination, inflammation, axonal damage, and virus distribution in Theiler’s murine encephalomyelitis (TME). Groups of two to six *glial fibrillary acidic protein (GFAP)-thymidine-kinase* transgenic *SJL* mice and *SJL* wildtype mice were infected with TME virus (TMEV) or mock (vehicle only). Astrocyte depletion was induced by the intraperitoneal administration of ganciclovir during the early and late phase of TME. The animals were clinically investigated while using a scoring system and a rotarod performance test. Necropsies were performed at 46 and 77 days post infection. Cervical and thoracic spinal cord segments were investigated using hematoxylin and eosin (H&E), luxol fast blue-cresyl violet (LFB), immunohistochemistry targeting Amigo2, aquaporin 4, CD3, CD34, GFAP, ionized calcium-binding adapter molecule 1 (Iba1), myelin basic protein (MBP), non-phosphorylated neurofilaments (np-NF), periaxin, S100A10, TMEV, and immunoelectron microscopy. The astrocyte depleted mice showed a deterioration of clinical signs, a downregulation and disorganization of aquaporin 4 in perivascular astrocytes accompanied by vascular leakage. Furthermore, astrocyte depleted mice showed reduced inflammation and lower numbers of TMEV positive cells in the spinal cord. The present study indicates that astrocyte depletion in virus triggered CNS diseases contributes to a deterioration of clinical signs that are mediated by a dysfunction of perivascular astrocytes.

## 1. Introduction

Astrocytes represent the most abundant cell type in the central nervous system (CNS), outnumbering neurons by four- to fivefold [1,2,3,4,5]. They are involved in synapse formation, propagation of action potentials, maintenance of blood-brain (BBB), as well as blood-spinal cord barrier (BSCB), extracellular matrix homeostasis, and antigen presentation [6,7,8,9,10,11]. Astrocytes maintain the function of excitatory synapses by secretion of glypicans like glypican 4 and 6, SPARC-like protein 1, as well as thrombospondin 1 and 2 or induce synapse phagocytosis by secretion of toxic factors, like complement components and neurotoxins [8,9,12]. Astrocyte end-feet at the capillary surface are an integral part of BBB and BSCB, ensuring and maintaining a neuro protective milieu, blood flow, glucose uptake, and H_2_0 homeostasis [1,11,13,14,15,16,17,18,19]. Astrocytes quickly respond to various types of CNS injury by changing their morphology and cell proliferation [1,20].

Depending on their morphology and molecular expression, profile astrocytes can be categorized into A1 and A2 subtypes [3]. A1 astrocytes are mainly localized in the gray matter and possess numerous, highly branched processes. Complement cascade components and cytokines, like interleukin 1α and tumor necrosis factor (TNF) produced by microglia, promote the differentiation of astrocytes into the A1 subtype. Furthermore, an activation of the nuclear factor kappa light-chain-enhancer of activated B cells (NFĸB) signaling pathway is assumed to promote A1 polarity [3]. During differentiation, A1 astrocytes lose several neuroprotective functions and increase the expression of toxic factors (e.g. complement components, neurotoxins), leading to neuronal damage, loss of synapses and an impairment of saltatory conduction downregulating excitatory potentials [6,9,12,15,21]. The majority of A2 astrocytes are located in the white matter. They show longer processes with a lower degree of ramification when compared to A1 astrocytes and their activation is assumed to be mediated by the Januskinase-Signal transducers and activators of transcription (JAK-STAT)-3 pathway [3,22,23]. A2 astrocytes have a rather neuroprotective role, promoting the survival and growth of neurons and supporting synaptic function [3,15,16].

Reactive astrogliosis includes a range of molecular, cellular, and functional changes, enabling astrocytes to respond to all forms and severities of CNS injury along a continuum of adaptive transcriptomic and proteomic changes, cellular hypertrophy, proliferation, and scar formation [1]. Astrocytes participate in the development and progression of demyelinating diseases, like multiple sclerosis (MS) [24]. Theiler’s murine encephalomyelitis (TME) is a well-established virus induced animal model for studying the pathogenesis of progressive MS [25,26,27,28,29,30]. After intracerebral infection of susceptible mice with a low neurovirulent TME virus (TMEV) strain (like BeAn), animals develop a biphasic disease that consists of an acute polioencephalitis, followed by a progressive demyelinating leukomyelitis with astrogliosis [25,29,31,32,33]. During the acute phase of TME, virus replication takes place in neurons, whereas the virus resides in oligodendrocytes and microglia/macrophages during the chronic phase [25,29,31,32,33,34]. Mechanisms leading to demyelination in the chronic phase of TME include delayed-type hypersensitivity, bystander demyelination, as well as virus mediated oligodendrocyte death [35,36,37]. The pathogenic role of virus specific T lymphocytes is still incompletely understood [38]. Whether the effects of TMEV upon lymphocytes and microglia/macrophages are primarily mediated by astrocytes has not been investigated yet. Since *in vitro* studies using TMEV infected astrocytes show an upregulation of numerous proinflammatory cytokines, including IL-12 and TNF [39], it can be speculated that astrocytes significantly contribute to immune cell activation.

In TME, astrocytes increase in number over time with a significant astrogliosis starting at 98 days post infection (dpi) [40]. Astrogliosis is associated with a significant lack of remyelination and similar findings are observed in other studies while using pharmacological and genetical depletion of astrocytes. Using a myelin oligodendrocyte glycoprotein-induced model of experimental autoimmune encephalomyelitis (EAE), astrocytic death resulted in a failure of tissue scar formation, increased immune cell infiltration, and worsened clinical score [4,41,42]. Studies investigating stab wounds and crush injuries show similar findings with a higher degree of cell death and increased inflammation [4,19]. However, few studies reported contradictory results as astrocyte depletion using a cuprizone-induced model resulted in reduced immune cell activation that was associated with less efficient myelin debris removal [4,7]. Furthermore, astrocyte depletion prior to EAE induction led to an increased number of T lymphocytes, while astrocyte depletion at EAE onset was associated with an increased number of macrophages [43].

The role of astrocytes during demyelinating diseases remains to be clarified, as both beneficial and detrimental effects are reported [1,3,6,7,44,45,46,47]. Therefore, the hypothesis of the present study was that astrocyte depletion would lead to an improvement of clinical signs that are associated with reduced inflammation and virus load. The aim was to investigate the impact of astrocytes depletion upon de- and remyelination, inflammation, axonal damage, and virus distribution in TME. In addition, the spatio-temporal aspect of astrocytes depletion upon disease progression was studied.

## 2. Results

### 2.1. Clinical Investigation

**Figure 1:**
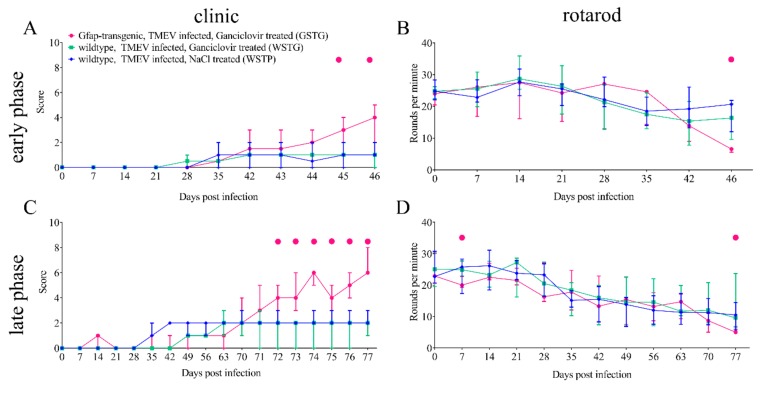
Clinical investigation using a scoring system and a rotarod test. Astrocyte depletion between 28–46 days post infection (dpi; early phase) resulted in a deterioration of clinical signs at 45 and 46 dpi (**A**) and rotarod performance at 46 dpi (**B**) in Theiler’s murine encephalomyelitis virus (TMEV) infected, ganciclovir treated *Gfap*-transgenic (GSTG) mice as compared with TMEV infected, ganciclovir treated wildtype mice (WSTG). Astrocyte depletion between 56–77 dpi (late phase) resulted in a deterioration of clinical signs starting at 72 dpi (**C**) and rotarod performance at 7 and 77 dpi (**D**) in GSTG as compared with WSTG mice. Clinical and rotarod data are shown as median ± standard deviation. Significant differences between GSTG and the other groups, as obtained by the Kruskal–Wallis test, followed by Mann–Whitney U post hoc tests were indicated by ●, *p* < 0.05.

Clinical scoring and rotarod performance tests were used to evaluate the impact of astrocyte depletion during TME (Figure 1). The observed clinical signs included reduced general condition, shaggy fur, waddling gait, and progressive ataxia starting at 45 dpi. Astrocyte depletion in the early phase (ganciclovir treatment between 28–46 dpi) resulted in a deterioration of clinical signs and rotarod performance in TMEV infected, ganciclovir treated *Gfap*-transgenic (GSTG) mice as compared with TMEV infected, ganciclovir treated wildtype mice (WSTG; Figure 1). Astrocyte depletion in the late phase (ganciclovir treatment between 56–77 dpi) resulted in a deterioration of clinical signs starting at 72 dpi and rotarod performance at 77 dpi in GSTG when compared with WSTG mice (Figure 1). Summarized, astrocyte depletion in the early and late phase was associated with a deterioration of clinical signs and rotarod performance.

### 2.2. Effects of Astrocyte Depletion upon TME

**Figure 2:**
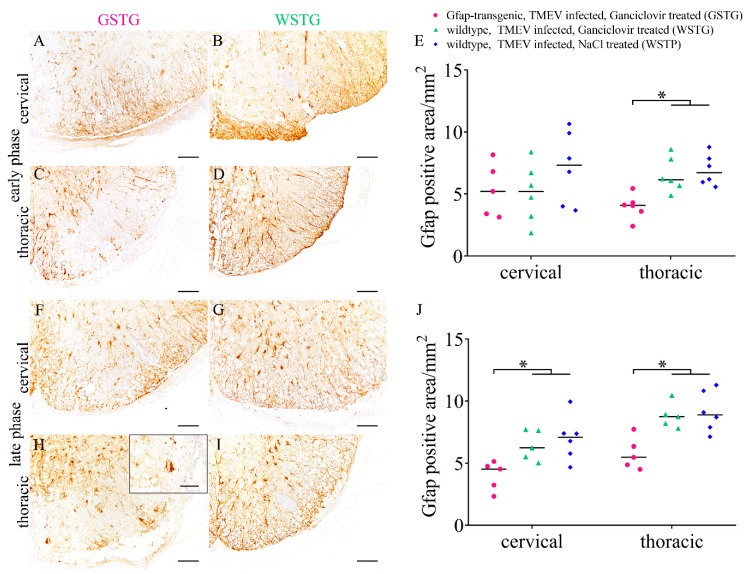
Astrocyte distribution during Theiler’s murine encephalomyelitis (TME). Immunohistochemistry targeting glial fibrillary acidic protein (GFAP) was used for the detection of astrocytes. At 46 days post infection (dpi; early phase) TMEV infected, ganciclovir treated, *Gfap*-transgenic mice showed a significantly reduced GFAP-positive area in the white matter of the thoracic spinal cord (**A**,**C**) compared to TMEV infected, ganciclovir treated wildtype (WSTG; **B**,**D**) and TMEV infected, natrium chloride treated wildtype (WSTP; **E**) mice. At 77 dpi (late phase) TMEV infected, ganciclovir treated, *Gfap*-transgenic mice showed a significantly reduced GFAP-positive area in the white matter of the cervical and thoracic spinal cord segment (**F**,**H**) compared with WSTG (**G**,**I**) and WSTP (**J**). Insert shows the morphology of an intralesional astrocyte in a GSTG animal (**H**). Data are shown as scatter dot plots. Significant differences between the groups obtained by Kruskal-Wallis test followed by Mann–Whitney U post hoc tests were indicated by *, *p* < 0.05. Bars represent 100 µm in overviews and 50 µm in the insert.

Astrocytes were quantified while using immunohistochemistry targeting glial fibrillary acidic protein (GFAP) to investigate their impact upon the progression of TME (Figure 2). Intralesional astrocytes in GSTG animals were enlarged and they showed a rounded shape with a limited number of processes (Figure 2). Ganciclovir treatment of GSTG mice between days 28 to 46 (early phase) led to a significant reduction of the GFAP positive area in the thoracic spinal cord segment, while the cervical segment was not affected (Figure 2). Ganciclovir treatment between days 56 to 77 (late phase) resulted in a significant reduction of the GFAP positive area in both spinal cord segments of GSTG mice (Figure 2).

### 2.3. Astrocyte Phenotype

**Figure 3:**
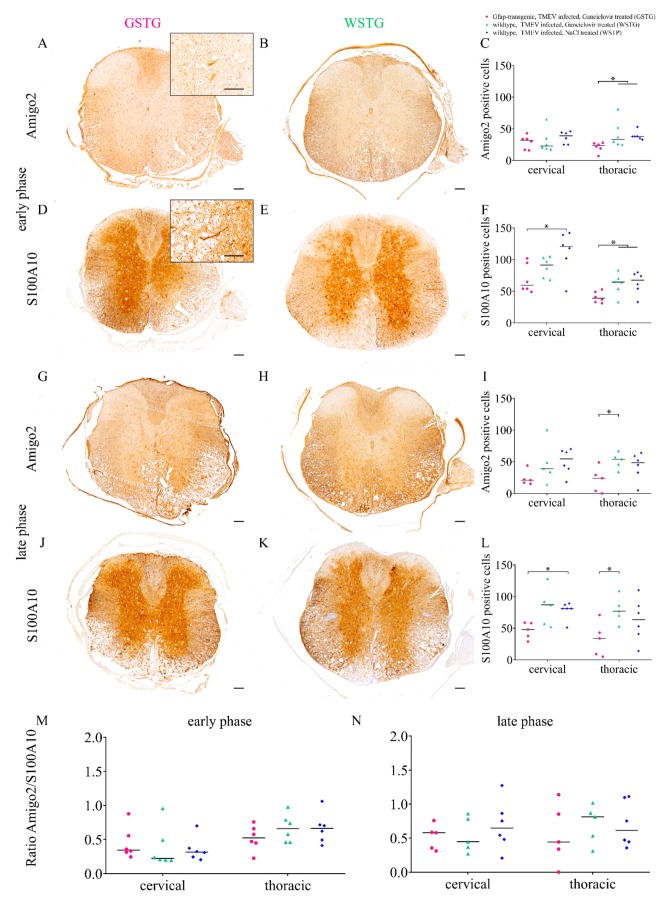
Astrocyte phenotype identification and quantification. Identification and quantification of astrocyte phenotypes in the ventral part of the spinal cord white matter were performed using immunohistochemistry identifying A1 (Amigo2, insert in **A**) and A2 (S100A10, insert in **D**). Astrocyte depletion resulted in a reduced number of Amigo2 positive cells in the thoracic spinal cord of TMEV infected, ganciclovir treated, *Gfap*-transgenic mice (GSTG) mice at 46 days post infection (dpi; **A**–**C**) and 77 dpi (late phase; **G–I**). Immunohistochemistry for S100A10 (A2 astrocytes) detected a significant reduction of the A2 subpopulation in the cervical and thoracic spinal cord at 46 dpi (early phase; **D**–**F**) and 77 dpi (late phase; **J**–**L**). Statistical analysis did not reveal a significant difference between the ratios of A1 and A2 astrocytes in all investigated groups. However, at 46 dpi (**M**) and 77 dpi (**N**) a variable predominance of A2 astrocytes in all groups was observed. Data are shown as scatter dot plots. Significant differences between the groups obtained by Kruskal–Wallis test followed by Mann–Whitney U post hoc tests were indicated by *, *p* < 0.05. Bars represent 100 µm in overviews and 50 µm in the inserts.

Immunohistochemistry identifying A1 (Amigo2) and A2 (S100A10) astrocyte subpopulations was performed since different astrocyte phenotypes are associated with beneficial or detrimental functions. Astrocyte depletion during the early (ganciclovir treatment between 28 and 46 dpi) and late phase (ganciclovir treatment between 56 and 77 dpi) was associated with a reduced number of Amigo2 positive cells in the thoracic spinal cord segment and of S100A10 positive cells in the cervical and thoracic spinal cord segment of GSTG mice (Figure 3). For the determination of the predominant astrocyte phenotype a ratio of A1 and A2 cells was calculated. Statistical analysis revealed no significant difference between the ratios of all the investigated groups. However, all groups showed a variable predominance of A2 when compared to A1 cells (Figure 3).

### 2.4. Quantification and Subcellular Localization of Aquaporin 4

**Figure 4:**
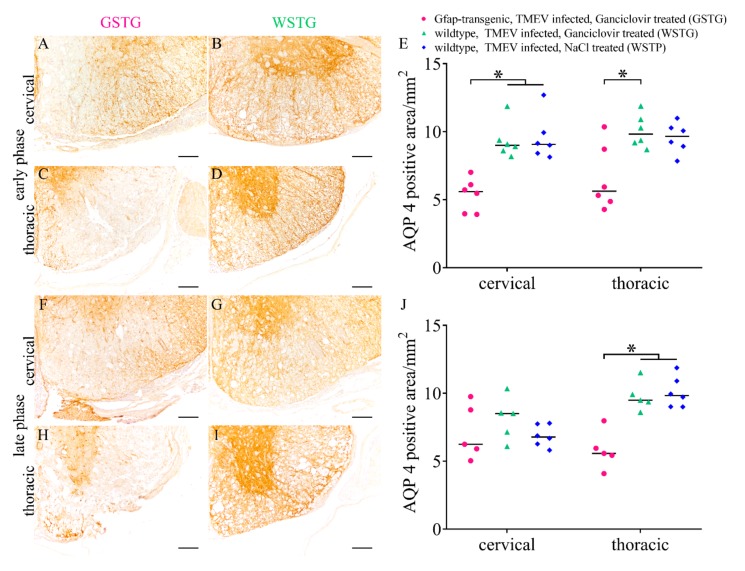
Impact of astrocyte depletion upon aquaporin 4 (AQP4) expression. Immunohistochemistry targeting AQP4 revealed a significant reduction of AQP4 positive area in the cervical and thoracic spinal cord segment of TMEV infected, ganciclovir treated, *Gfap*-transgenic mice (GSTG) mice at 46 days post infection (dpi; early phase; **A**–**E**). At 77 dpi (late phase), GSTG mice showed a significant reduction of AQP4 positive area in the thoracic spinal cord (**F**–**J**). Data are shown as scatter dot plots. Significant differences between the groups obtained by Kruskal-Wallis test followed by Mann–Whitney U post hoc tests were indicated by *, *p* < 0.05. Bars represent 100 µm.

The expression of the water channel protein aquaporin 4 (AQP4) was investigated while using immunohistochemistry (Figure 4). Statistical analysis showed a significant reduction of AQP4 in the cervical and thoracic spinal cord segment of GSTG mice at 46 dpi. At 77 dpi a reduction of AQP4 in the thoracic spinal cord segment of GSTG mice was detected. Summarized, immunohistochemistry targeting AQP4 revealed a reduction of the AQP4 positive area in GSTG mice.

**Figure 5:**
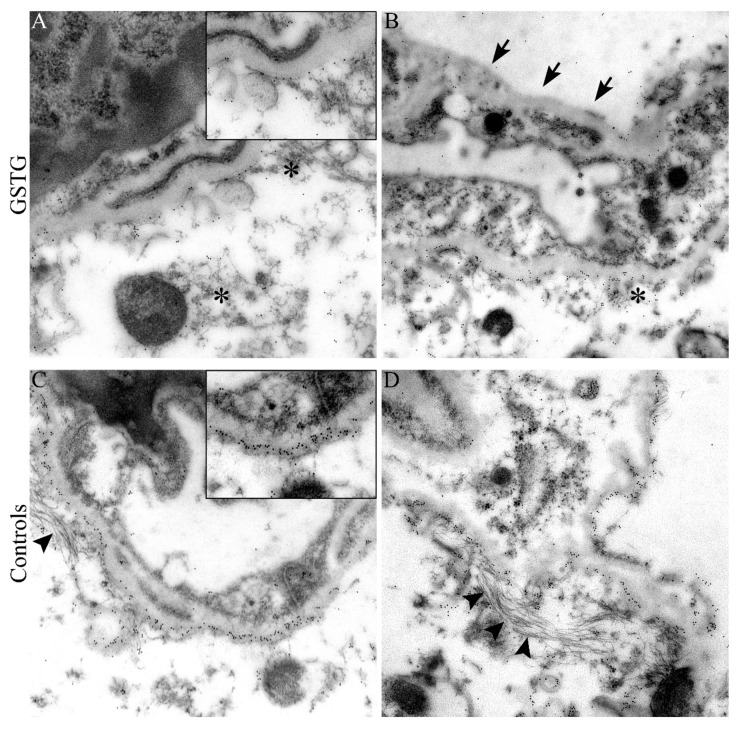
Immunoelectron microscopy targeting aquaporin 4 (AQP4). Immunoelectron microscopy of the thoracic spinal cord segment was performed for the localization of AQP4 within perivascular astrocytes. A reduced amount and an irregular distribution of AQP4 protein along the cytoplasmic membrane of perivascular astrocyte end-feet (arrows in **B**) in TMEV infected, ganciclovir treated, *Gfap*-transgenic (GSTG, **A**,**B**) mice compared to control mice (**C**,**D**) at 77 days post infection was detected. The distribution of AQP4 along the cytoplasmic membrane is shown in higher magnification (inserts in **A**,**C**). The reduced expression of AQP4 in GSTG animals was associated with a shortening and disorganization of intermediate filaments (asterisks in **A**,**B**) in comparison to control animals (arrowheads in **C**,**D**). Magnifications are 40,000× in **A**, 25,000× in **B** and 31,500 in **C**,**D**.

Immunoelectron microscopy was performed to substantiate this finding and to elucidate the distribution and localization of AQP4 in perivascular astrocytes contributing to the BSCB. Immunolabeling of AQP4 revealed a reduced number and irregular frequency of gold particles along the cytoplasmic membrane of perivascular astrocyte end-feet in the thoracic spinal cord segment of GSTG mice (Figure 5). Moreover, associated with the irregular arrangement of AQP4 along the cytoplasmic membrane a shortening and disorganization of intermediate filaments in GSTG mice was observed (Figure 5).

### 2.5. Detection of Vascular Leakage

**Figure 6:**
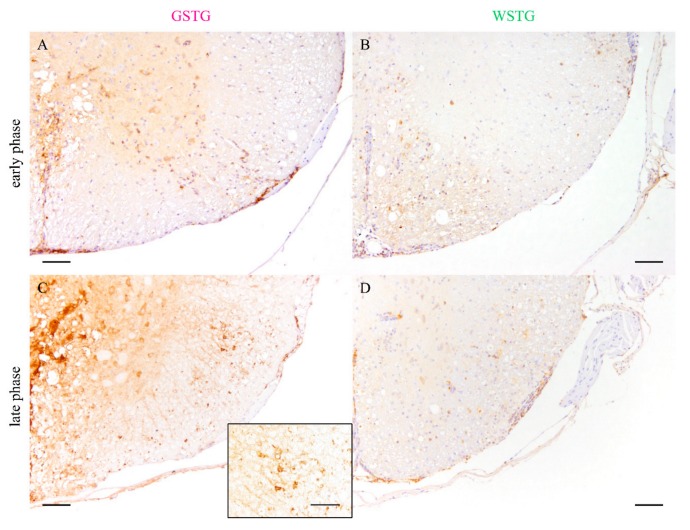
Detection of vascular leakage. Immunohistochemistry targeting CD34 was used for the detection of vascular leakage within the spinal cord white matter. Variable CD34 labeling was predominantly observed in the ventral part of the cervical and thoracic spinal cord white matter at 46 (early phase; **A**,**B**) and 77 (late phase; **C**,**D**) days post infection in all groups. CD34 labeling is shown in higher magnification (insert in **C**). Bars represent 100 µm in the overviews and 50 µm in the insert.

Vascular leakage within the white matter was detected while using immunohistochemistry targeting CD34. Scattered and accentuated in the ventral part of the cervical and thoracic spinal cord CD34 expression was detected with variable degree in all groups at 46 and 77 dpi (Figure 6).

### 2.6. Quantification of Inflammation and Immunophenotyping of Inflammatory Cells

**Figure 7:**
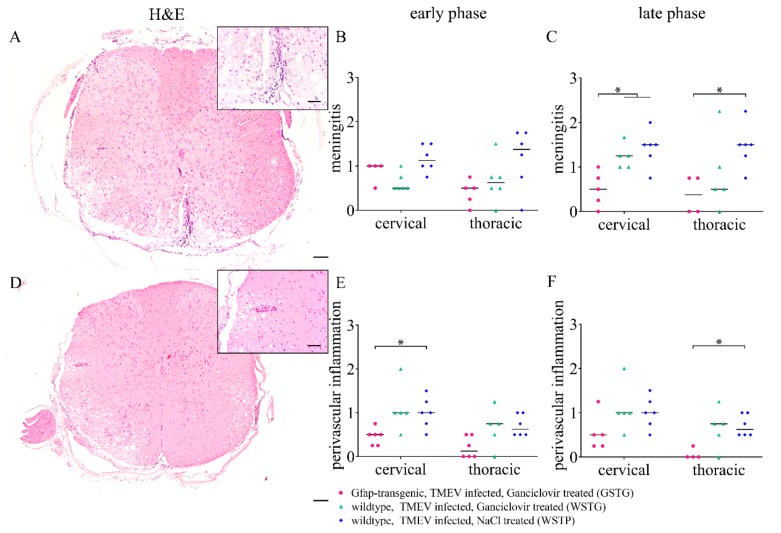
Quantification of meningeal and perivascular inflammation. Meningitis and perivascular inflammation in the cervical and thoracic spinal cord white matter were quantified using hematoxylin and eosin (H&E) staining. Statistical analysis revealed a significant reduction of meningitis in TMEV infected, ganciclovir treated, *Gfap*-transgenic (GSTG) mice at 77 days post infection (dpi; late phase; **A**–**C**). Perivascular inflammation in the white matter of the cervical spinal cord at 46 dpi (early phase; **E**) and of the thoracic spinal cord at 77 dpi (late phase; **F**) was significantly reduced in GSTG animals. The inserts in **A** and **D** show higher magnifications. Data are shown as scatter dot plots. Significant differences between the groups obtained by Kruskal–Wallis test followed by Mann–Whitney U post hoc tests were indicated by *, *p* < 0.05. Bars represent 100 µm in the overviews and 50 µm in the inserts.

Scoring of meningitis and perivascular inflammation in the cervical and thoracic spinal cord white matter was performed to investigate the impact of astrocyte depletion upon inflammation (Figure 7). At 77 dpi, a reduced degree of meningitis in the cervical and thoracic spinal cord of GSTG mice was detected (Figure 7). Perivascular inflammation was significantly reduced in GSTG mice in the cervical white matter at 6 dpi and in the thoracic white matter at 77 dpi.

**Figure 8:**
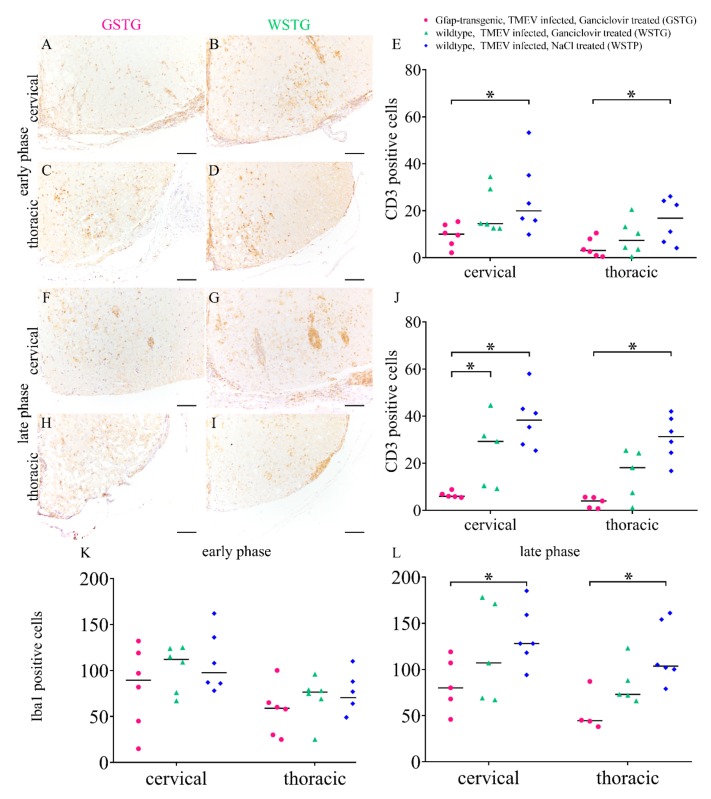
Immunophenotyping and quantification of inflammatory cells. T lymphocytes (CD3) were quantified in the cervical and thoracic spinal cord using immunohistochemistry. TMEV infected, ganciclovir treated, *Gfap*-transgenic (GSTG) mice showed a reduced number of CD3 positive cells in the cervical and thoracic spinal cord at 46 days post infection (dpi; early phase; **A**–**E**) and 77 dpi (late phase; **F**–**J**). Immunohistochemistry targeting ionized calcium-binding adapter molecule 1 (Iba1) was used for the quantification of microglia/macrophages. While at 46 dpi no significant differences between the groups were detected (**K**) at 77 dpi the number of Iba1 labeled cells in the cervical and thoracic spinal cord was significantly reduced in GSTG mice (**L**). Data are shown as scatter dot plots. Significant differences between the groups obtained by Kruskal–Wallis test followed by Mann–Whitney U post hoc tests were indicated by *, *p* < 0.05. Bars represent 100 µm.

Immunophenotyping of inflammatory cells included T lymphocytes (CD3) and microglia/macrophages (ionized calcium-binding adapter molecule 1, Iba1). CD3 immunohistochemistry revealed a reduced number of CD3 positive cells in the cervical and thoracic spinal cord of GSTG mice as compared to control animals at all investigated time points (Figure 8). While no significant differences between the numbers of Iba1 positive cells were detected at 46 dpi, the number of Iba1 positive cells was significantly reduced in the cervical and thoracic spinal cord segment of GSTG mice at 77 dpi (Figure 8).

### 2.7. Impact of Astrocyte Depletion upon Demyelination, Schwann Cell Remyelination, Axonal Damage and Virus Protein

**Figure 9:**
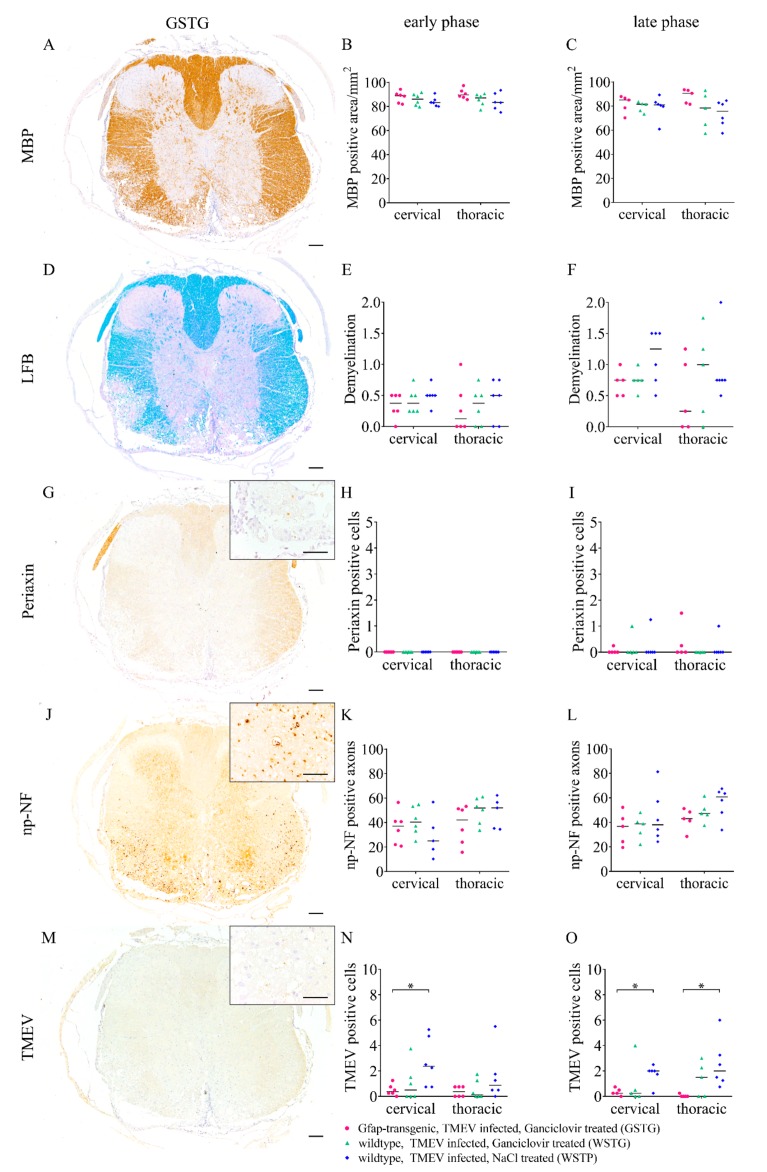
Quantification of demyelination, Schwann cell remyelination, axonal damage, and virus protein. Myelin basic protein (MBP; **A–C**) immunohistochemistry and luxol fast blue-cresyl violet (LFB; **D**–**F**) staining were used to quantify demyelination after astrocyte depletion. Accentuated in the ventral parts of the spinal cord white matter demyelination occurred in a variable degree in all groups. However, statistical analysis did not reveal a significant difference between TMEV infected, ganciclovir treated, *Gfap*-transgenic (GSTG) mice and the other groups (**B**,**C**,**E**,**F**). Periaxin immunohistochemistry was used to detect Schwann cell remyelination (**G**–**I**). At 77 days post infection (dpi; late phase) the cervical and thoracic spinal cords showed low numbers of Schwann cells in all groups (I). However, statistical analysis did not reveal a significant difference of periaxin positive Schwann cell numbers in GSTG mice compared to the control groups (**H**,**I**). Immunohistochemistry detecting non-phosphorylated-neurofilaments (np-NF) was used to detect and quantify axonal damage (**J**–**L**). Accentuated in the ventral parts of the white matter variable numbers of np-NF labeled axons were detected in all groups. Statistical analysis did not reveal a significant difference of axonal damage in GSTG mice compared to the controls at 46 dpi (early phase; K) and 77 dpi (late phase; **L**). Virus protein was detected and quantified by Theiler’s murine encephalomyelitis virus (TMEV) immunohistochemistry (**M**–**O**). Virus positive cells were present in the cervical and thoracic spinal cord of all groups. GSTG mice revealed a significantly reduced number of TMEV positive cells in the cervical spinal cord at 46 dpi (early phase, **N**) and in the cervical and thoracic spinal cord at 77 dpi (late phase; **O**). The data are shown as scatter dot plots. Significant differences between the groups obtained by Kruskal-Wallis test followed by Mann–Whitney U post hoc tests were indicated by *, *p* < 0.05. Bars represent 100 µm in the overviews and 50 µm in the inserts.

Demyelination of the white matter was evaluated while using immunohistochemistry targeting myelin basic protein (MBP) and luxol fast blue-cresyl violet (LFB) staining (Figure 9). Accentuated in the ventral part of the cervical and thoracic spinal cord white matter demyelination was detected in all groups. Statistical analysis revealed no significant differences between GSTG and the other groups at all the investigated time points.

Schwann cell remyelination was quantified while using immunohistochemistry targeting periaxin (Figure 9). Low numbers of Schwann cells were detected in the ventral part of the cervical and thoracic spinal cord in all groups at 77 dpi. However, statistical analysis revealed no significant difference in the number of periaxin labeled cells between the groups.

Axonal damage was investigated by counting non-phosphorylated-neurofilaments (np-NF) positive axons (Figure 9). Variable numbers of np-NF labeled axons were found in the spinal cord white matter of all groups. Statistical analysis did not detect a significant difference in the number of immunolabeled axons between the groups at all of the investigated time points.

Furthermore, TMEV protein was visualized and quantified (Figure 9). Virus positive cells were detected in the ventral part of the spinal cord white matter in animals of all groups and at all investigated time points. GSTG animals showed a significantly reduced number of TMEV positive cells in the cervical spinal cord at 46 dpi and in the cervical and thoracic spinal cord at 77 dpi.

### 2.8. Effects of Ganciclovir Treatment in Mock Infected Mice

Mock infected *SJL.Cg-Tg(Gfap-TK)^7.1Mvs^* and *SJL/JOlaHsd* mice with ganciclovir treatment did not show significant differences in the clinical investigations as well as by using immunohistochemistry targeting aquaporin 4, CD3, GFAP, Iba1, MBP, np-NF, and periaxin.

## 3. Discussion

**Figure 10:**
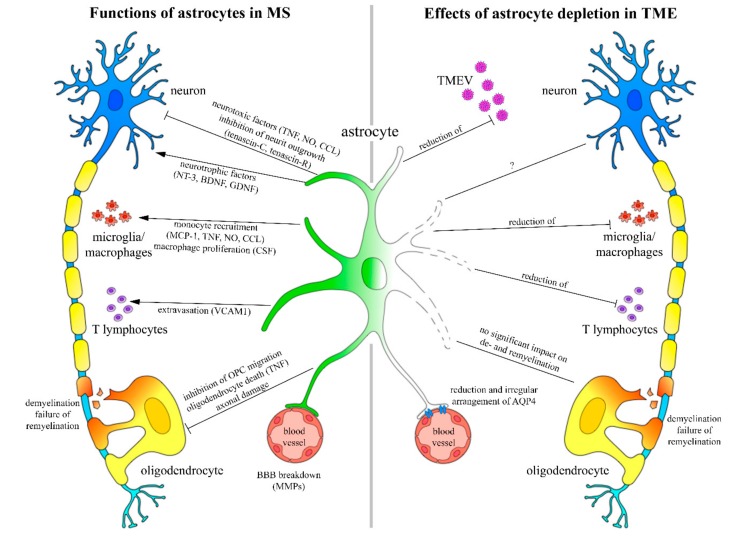
Presumed roles of astrocytes in multiple sclerosis (MS) and Theiler’s murine encephalomyelitis. Functions of astrocytes in MS are shown on the left side of the scheme [8,48,49,50,51]. They include an interaction with neurons by secreting neurotrophic as well as neurotoxic factors. Microglia/macrophage recruitment and T lymphocyte extravasation are induced by astrocytes as well. Furthermore, astrocytes are able to inhibit oligodendrocyte precursor cell (OPC) migration and induce oligodendrocyte death. Secretion of matrix metalloproteinases (MMPs) like MMP-9 significantly contributes to an impairment of the blood brain barrier (BBB). The right side of the schematic drawing summarizes the effects of astrocyte depletion in Theiler’s murine encephalomyelitis (TME). Astrocyte depletion during the early and late phase of TME resulted in a lower number of TMEV positive cells and a reduction of spinal cord inflammation with no effect on de- and remyelination. Furthermore, astrocyte depletion was associated with a reduction and an irregular arrangement of aquaporin 4 (AQP4) water channels, as well as a shortening and disorganization of intermediate filaments in perivascular astrocytes. BDNF, Brain-derived neurotrophic factor; CCL, chemokine ligand; GDNF, Glial cell line-derived neurotrophic factor; NO, nitric oxide; NT-3, Neurotrophin-3; TNF, tumor necrosis factor.

Glia cells, including oligodendrocytes, microglia, and inactive as well as resting astrocytes represent a frequent and important cell type in the CNS [6,16]. CNS injury triggers astrocyte activation, proliferation, and finally the formation of a glial scar in direct proximity to the insult. Astrocytes are able to react fast and specifically by changing their morphological, antigenic, and physiological characteristics, depending on the localization as well as the severity and type of the insult [1,4,5]. Astrocytes play a crucial role in demyelinating diseases, including MS and TME (Figure 10).

They contribute to neuronal plasticity, including synapse formation, by secreting various neurotrophic and neurotoxic factors (Figure 10) [9]. Furthermore, astrocytes interfere with the adaptive immune response by regulating monocyte recruitment, extravasation, and proliferation [8,44,45]. Demyelination is one of the hallmarks in MS. Major factors leading to demyelination include T lymphocyte mediated immunity, T lymphocyte and antibody mediated immunity, as well as an oligodendrocyte dystrophy [46]. Astrocytes contribute to demyelination by inhibiting oligodendrocyte precursor cell (OPC) migration and differentiation, as well as inducing oligodendrocyte death [6,42,47]. In MS and TME, a block of OPC differentiation is suggested to cause insufficient remyelination [30,48,49]. Additionally, neuronal activity regulates myelination and remyelination by an intense axon-OPC interaction that is partly mediated by glutamate [50,51,52]. The hypothesis of the present study was that astrocyte depletion would lead to an improvement of clinical signs that are associated with reduced inflammation and virus load since TME represents a well-established animal model for the progressive forms of MS [28].

In this study, astrocyte depletion induced a deterioration of clinical signs and rotarod performance. An overall reduction of the GFAP positive area was associated with a change in the morphology of the astrocytes. Intralesional astrocytes were enlarged, showing a rounded shape and a limited number of processes. An evaluation of astrocytes using immunoelectron microscopy revealed an irregular arrangement and shortening of intermediate filaments.

Astrocytes are considered to have either a beneficial or a detrimental effect in neuroinflammation [3,18]. In the present study astrocyte depleted mice showed a reduced number of TMEV positive cells as well as a reduced degree of meningitis and perivascular inflammation. The immunophenotyping of inflammatory cells revealed a reduced number of CD3 positive T lymphocytes and Iba1 labeled microglia/macrophages within the spinal cord white matter (Figure 10). The contribution of astrocyte depletion to disease progression was previously investigated in other animal models of MS like EAE and cuprizone induced demyelination [7,43]. Astrocyte depletion in EAE leads to an increased severity of disease that is associated with increased inflammation mediated by macrophages but not T lymphocytes [43]. The major effect of astrocyte depletion in cuprizone induced demyelination was a reduced microglia/macrophage activation, leading to the persistence of myelin debris and reduced remyelination [7]. However, a comparative study using TME, EAE, and cuprizone induced demyelination showed that the drug ganciclovir has no impact upon microglia numbers, proliferation rates, or cellular viability [52].

Therefore, the observed reduced degree of inflammation in the present study might be attributed to a reduced activation of microglia/macrophages by astrocytes. Important pathways for astrocyte and microglia/macrophage or T lymphocyte activation include antigen presentation using major histocompatibility complex class II (MHCII) on astrocytes as well as chemokines, like Chemokine (C–X–C motif) ligand 10 [7] and numerous cytokines that are secreted by astrocytes, like IL-12, IL-23, and IL-15, promoting T lymphocyte differentiation [6,18]. However, reduced inflammation in astrocyte depleted mice was not associated with reduced axonal damage, demyelination, or more prominent remyelination.

Astrocytes are classified into different subpopulations, namely A1 and A2 [3,9]. A1 astrocytes are mainly found in inflammatory lesions and they are considered as the more harmful subtype inducing neuronal damage, loss of synapses, and impaired saltatory action potential propagation [9,12]. In contrast, A2 astrocytes are postulated to be more neuroprotective [3,9]. To analyze the effect of astrocyte depletion upon the deterioration of clinical signs immunohistochemistry identifying A1 and A2 astrocyte subpopulations was performed. The astrocyte depleted mice showed a reduction of the number of A1 and A2 astrocyte subpopulations at both investigated time points (46 and 77 dpi). The ratio of both subpopulations indicated a higher abundance of A2 as compared to A1 astrocytes. Whether this finding has a direct impact upon saltatory conduction and synapse plasticity explaining the observed deterioration of clinical signs needs to be evaluated in future studies.

The integrity of the blood-spinal cord barrier is essential for creating and maintaining ionic balance as well as water homeostasis [14,15]. Small lipophilic molecules like oxygen and carbon dioxide are able to diffuse freely through lipid membranes, while larger particles like water molecules (H_2_O) need an active transport [14]. Water channel proteins, like AQP4, regulate H_2_O transport across lipid membranes that are driven by an osmotic gradient [1,13,53,54]. Previous publications showed a presence of anti-AQP4 antibodies during the inflammatory and demyelinating phase of neuromyelitis optica, which indicated that this molecule might represent a specific target in demyelinating diseases of the CNS [55,56]. Furthermore, studies in EAE confirmed the relationship between AQP4 and neuroinflammation [56,57]. In the present study, astrocyte depleted mice showed a significant reduction of the AQP4 positive area at 46 and 77 dpi. Ultrastructural analysis revealed a reduced number and irregular frequency of AQP4 channels along the cytoplasmic membrane of perivascular astrocytes in astrocyte depleted mice, which was associated with a shortening and disorganization of intermediate filaments (Figure 10).

Previous studies show that AQP4 is involved in astrocyte cytoskeleton organization and cell plasticity by an interaction with connexin 43 [58,59]. Therefore, the observed alterations in astrocyte shape and size in the present study are considered to be attributed to the reduced and irregular expression of AQP4. However, whether AQP4 directly affects the cytoskeletal organization via an interaction with intermediate filaments like GFAP or this effect represents a consequence of volume changes and transcriptional alterations remain to be determined [58].

CNS function is strongly coupled to an efficient control of extracellular volume mainly driven by astrocytes [13]. Especially water channels like AQP4 are responsible for basic and dynamic volume changes and participate in excitatory synapse function [13,60]. Furthermore, perivascular astrocyte end-feet contain a 10-fold higher density of AQP4 than non-end-feet membranes [13,53]. Downregulation and a disorganized expression of AQP4 change the composition of the extracellular matrix and can affect the diffusion and clearance of signal molecules [13,61]. In the present study, CD34 was used for the visualization of vascular leakage within the spinal cord white matter [62]. Vascular leakage was detected in all TMEV infected groups, with variable degree. However, the reduced and disorganized expression of AQP4 in perivascular astrocytes was coherent with alterations in astrocyte morphology and it may have resulted in a different composition of the extracellular matrix, which has contributed to the deterioration of clinical signs in astrocyte depleted animals.

## 4. Materials and Methods

### 4.1. Study Design and Animals

For animal experiments, two to six female, five to six week-old *SJL.Cg-Tg(Gfap-TK)^7.1Mvs^* and *SJL/JOlaHsd* mice were used. The animals were randomly assigned to treatment groups while using a randomization sheet designed in Microsoft™ Excel. Mice were housed in groups of 2 to 6 individuals in isolated ventilated cages (Tecniplast, Hohenpeißenberg, Germany) in a standardized environment (22.5 ± 1.5 °C, 55 ± 10% relative humidity, 12 h light/dark cycle, 74 changes of air per hour), fed a standard rodent diet (R/M-H; Ssniff Spezialdiäten GmbH, Soest, Germany) ad libitum and had free access to water.

### 4.2. Theiler Virus Infection and Ganciclovir Treatment

**Figure 11:**
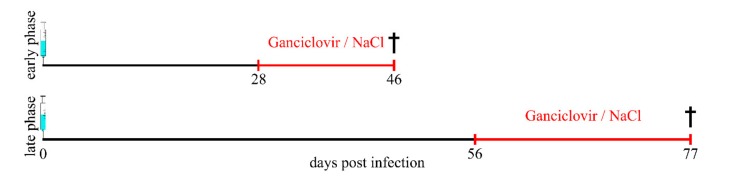
Study design. Groups of 2–6 *GFAP-thymidine-kinase* transgenic *SJL* mice and *SJL* wildtype mice were infected with Theiler’s murine encephalomyelitis virus (TMEV) or mock (vehicle only). Astrocyte depletion was induced by once daily intraperitoneal administration of ganciclovir from 28 to 46 (early phase) or 56 to 77 (late phase) days post infection. Red line indicates the ganciclovir/NaCl treatment period.

Intracerebral injection in the right hemisphere either with TMEV (1.14 × 10^5^ plaque-forming units per mouse, BeAn strain, passage 3) or mock (vehicle only) was performed in general anesthesia, as previously described [63]. For anesthesia, mice received an injection of ketamine (100 mg/kg Ketamin 10%, WDT, Garbsen, Germany) and medetomidine (0.5 mg/kg, Domitor^®^, Pfizer, Berlin, Germany). The animals received once daily an intraperitoneal injection of either ganciclovir (25 mg/kg, Cymevene™, Par Sterile Product LLC, Chestnut Ridge, NY, USA) diluted in 0.9% sodium chloride solution (WDT, Garbsen, Germany) or 0.9% sodium chloride solution only (WDT, Garbsen, Germany), from 28 to 46 (early phase) or 56 to 77 (late phase) days post TMEV infection (Figure 11).

### 4.3. Clinical Examination and Rotarod Performance

The clinical examination of the animals included a scoring system evaluating posture and physical appearance (0–3), behavior and activity (0–3), as well as gait (0–4), with a 0 indicating normal. All of the scores per animal were added to a total score. Rotarod performance test (RotaRod treadmill, TSE Technical & Scientific Equipment, Bad Homburg, Germany) was performed once a week. Animals were trained at five and three days before infection for 10 min with a constant speed of 5 rpm (rounds per minute) or 10 rpm, respectively. During the experiment, an accelerated rotarod test (rotation speed was increased from 5 to 55 rpm over a duration of five minutes) was used. For each animal, the test was repeated three times and the mean value was used for statistical analysis.

### 4.4. Tissue Sampling and Preparation

The animals were perfused with phosphate buffered saline (PBS, pH 7.4). Cervical (C1-3) and thoracic (T3-5) spinal cord were removed from the vertebral column and then fixed in formalin. In addition, thoracic spinal cord (T7-12) was mounted in Tissue-Tek O.C.T.™ Compound (Sakura, Alphen aan den Rijn, Netherlands) and snap frozen in liquid nitrogen.

### 4.5. Histochemistry and Immunohistochemistry

For histological examination, formalin-fixed samples were embedded in paraffin wax. The serial sections of the spinal cord segments were prepared and stained with hematoxylin and eosin (H&E) as well as LFB. One investigator blinded to the treatment condition evaluated all sections. Semiquantitative evaluation was performed for meningitis, perivascular inflammation of white matter (0 = normal, 1 = single perivascular inflammation, 2 = two or three layers of inflammation, 3 = more than three layers of inflammation), and demyelination (0 = normal, 1 ≤ 25%, 2 = 26–50%, 3 ≥ 50%).

Immunohistochemistry was performed as previously described [28,30,63]. Applied antibodies, dilutions, and pretreatments are detailed in Table 1. Antigen-antibody reactions were visualized while using the avidin-biotin-peroxidase reagent (Vectastain, ABC Kit, Vector Laboratories, Burlingame, CA, United States) and 3,3′-Diaminobenzidine tetrahydrochloride (Sigma-Aldrich, St. Louis, MO, United States).

The quantification of TMEV, A1 astrocytes (AMIGO2), A2 astrocytes (S100A10), T lymphocytes (CD3), microglia/macrophages (Iba1), Schwann cells (periaxin) and axonal damage (np-NF) was performed by manually counting immunolabeled cells for each cross section. Vascular leakage was detected by CD34 immunohistochemistry.

For the quantification of GFAP, MBP, and AQP4 pictures of the cervical and thoracic spinal cord segment were taken with a photo microscope (BZ-9000E, Keyence Deutschland GmbH, Neu-Isenburg, Germany). The regions of interest (ROI) were outlined and measured by digital image analysis (analySIS^®^, Soft Imaging System GmbH, Münster, Germany).

### 4.6. Immunoelectron Microscopy

AQP4 distribution and density was investigated in perivascular astrocytes using immunogold labeling followed by transmission electron microscopy. The O.C.T. compound embedded frozen tissue samples of the thoracic spinal cord were cut with a cryotome (Leica CM 1950, Leica Biosystems, Nussloch, Germany) into 30 μm thick sections, fixed in paraformaldehyde (Merck KGaA, Darmstadt, Germany), and diluted in cacodylate buffer (Serva Electrophoresis GmbH, Heidelberg, Germany) for one night followed by embedding in LR-White (LR-White Resin, MEDIUM GRADE Acryl Resin, London Resin Company Ltd., Reading, United Kingdom). Ultrathin sections were prepared and immunolabeled with an anti-AQP4 antibody (1:50 dilution; Merck Millipore, Danvers, Massachusetts, USA), followed by a goat anti-Rabbit IgG 10nm Immunogold conjugate secondary antibody (BBI Solutions, Crumlin, United Kingdom). The sections were contrasted with uranyl acetate and lead citrate and then evaluated on a transmission electron microscope (EM 10A, Carl Zeiss Microscopy GmbH, Jena, Germany).

### 4.7. Statistical Analysis

Statistical analysis was performed while using the IBM SPSS™ program (version 21, New York, NY, USA) for Windows™. Data analysis included the Kruskal–Wallis tests, followed by Mann–Whitney U posthoc tests. Data were considered as statistical significant at *p*-value ≤ 0.05. Graphs were generated while using the GraphPad Prism program (GraphPad Software, San Diego, CA, USA) for Windows™.

### 4.8. Ethics Statement

Animal experiments were performed according to the German Animal Welfare Law and approved by the local authorities (Niedersächsisches Landesamt für Verbraucherschutz und Lebensmittelsicherheit, (LAVES) Oldenburg, Germany, permission number: 33.12-42502-04-12/0949).

## 5. Conclusions

The present study shows that astrocyte depletion in the early and late phase of TME leads to a deterioration of clinical signs, a downregulation of AQP4, vascular leakage, and reduced spinal cord inflammation. Reduced and disorganized expression of AQP4 was associated with alterations in size and morphology of perivascular astrocytes and it may have contributed to the deterioration of clinical signs by modifying the composition of the extracellular matrix. The explicit mechanisms leading to the clinical deterioration of astrocyte depleted mice remain elusive. Therefore, further studies should investigate the impact of astrocyte depletion upon the composition of the extracellular matrix influencing intercellular communication and neuroinflammation.

## Figures and Tables

**Table 1:** Primary antibodies, pretreatment, and dilutions used for immunohistochemistry.

Antigen	Pretreatment	Dilution	Clonality	Supplier	Catalog Number
Amigo2	0.5% triton	1:200	polyclonal rabbit	Bioss Antibodies Inc., Woburn, Massachusetts, USA	ABIN138654
AQP4	/	1:200	polyclonal rabbit	Merck Millipore, Danvers, Massachusetts, USA	AB 3594
CD3	microwave/ citrate buffer	1:250	polyclonal rabbit	DakoCytomation GmbH, Hamburg, Germany	A0452
CD34	microwave/ citrate buffer	1:50	monoclonal mouse	BDBioscience, Heidelberg, Germany	550390
GFAP	/	1:1000	polyclonal rabbit	DakoCytomation GmbH, Hamburg, Germany	Z0334
Iba1	/	1:1000	polyclonal rabbit	Wako Chemicals Inc., Richmond, VA, USA	PN 019-19741
MBP	/	1:500	polyclonal rabbit	Chemicon, Temecula, CA, USA	AB980
np-NF	microwave/ citrate buffer	1:20000	polyclonal rabbit	Covance, Los Angeles, CA, USA	SMI-311R
Periaxin	microwave/ citrate buffer	1:5000	polyclonal rabbit	Sigma-Aldrich Chemie GmbH, Taufkirchen, Germany	HPA001868
S100A10	/	1:100	monoclonal rabbit	Bio-Techne GmbH, Wiesbaden, Germany	Ab JF0987
TMEV	/	1:2000	polyclonal rabbit	Kummerfeld et al. [64]	/

## Abbreviations

ABC	Avidin-biotin-peroxidase complex method
AQP4	Aquaporin 4
BBB	Blood-brain barrier
BDNF	Brain-derived neurotrophic factor
BSCB	Blood-spinal cord barrier
CCL	Chemokine ligand
CNS	Central nervous system
CSF	Colony-stimulating factor
dpi	Days post infection
EAE	Experimental autoimmune encephalomyelitis
GDNF	Glial cell line-derived neurotrophic factor
GFAP	Glial fibrillary acidic protein
GSTG	*Gfap*-transgenic, TMEV infected, ganciclovir treated
H&E	Hematoxylin and eosin
Iba1	Ionized calcium-binding adapter molecule 1
IL	Interleukin
LFB	Luxol fast blue-cresyl violet
MBP	Myelin basic protein
MCP-1	Monocyte chemotactic protein 1
MHCII	Major histocompatibility complex class II
MMPs	Matrix metalloproteinases
MS	Multiple sclerosis
NFĸB	Nuclear factor kappa light-chain-enhancer of activated B cells
np-NF	Non-phosphorylated-neurofilaments
NT-3	Neurotrophin-3
OPC	Oligodendrocyte precursor cell
PBS	Phosphate buffered saline
ROI	of interest
rpm	Rounds per minute
SJL	Swiss Jim Lambert
TME	Theiler’s murine encephalomyelitis
TMEV	Theiler’s murine encephalomyelitis virus
TNF	Tumor necrosis factor
VCAM1	Vascular cell adhesion molecule 1
WSTG	Wildtype, TMEV infected, ganciclovir treated
WSTP	Wildtype, TMEV infected, natrium chloride treated
